# AutomiG, a Biosensor to Detect Alterations in miRNA Biogenesis and in Small RNA Silencing Guided by Perfect Target Complementarity

**DOI:** 10.1371/journal.pone.0074296

**Published:** 2013-09-03

**Authors:** Clément Carré, Caroline Jacquier, Anne-Laure Bougé, Fabrice de Chaumont, Corinne Besnard-Guerin, Hélène Thomassin, Josette Pidoux, Bruno Da Silva, Eleftheria Chalatsi, Sarah Zahra, Jean-Christophe Olivo-Marin, Hélène Munier-Lehmann, Christophe Antoniewski

**Affiliations:** 1 Drosophila Genetics and Epigenetics, Laboratory of Developmental Biology, CNRS UMR7622, Université Pierre et Marie Curie, Paris, France; 2 Institut Pasteur, Drosophila Genetics and Epigenetics, Paris, France; 3 Chromatin and Cell Biology, Institute of Human Genetics, Montpellier, France; 4 mRNA Regulation and Development, Institute of Human Genetics, Montpellier, France; 5 Institut Pasteur, Quantitative Image Analysis, Paris, France; 6 Inserm U1016 - CNRS UMR 8104, Faculté de Médecine-Cochin, Paris, France; 7 Institut Pasteur, Unité de Chimie et Biocatalyse, Département de Biologie Structurale et Chimie, Paris, France; 8 CNRS, UMR3523, Paris, France; Cardiff University, United Kingdom

## Abstract

Defects in miRNA biogenesis or activity are associated to development abnormalities and diseases. In *Drosophila*, miRNAs are predominantly loaded in Argonaute-1, which they guide for silencing of target RNAs. The miRNA pathway overlaps the RNAi pathway in this organism, as miRNAs may also associate with Argonaute-2, the mediator of RNAi. We set up a gene construct in which a single inducible promoter directs the expression of the GFP protein as well as two miRNAs perfectly matching the GFP sequences. We show that self-silencing of the resulting automiG gene requires Drosha, Pasha, Dicer-1, Dicer-2 and Argonaute-2 loaded with the anti-GFP miRNAs. In contrast, self-silencing of the automiG gene does not involve Argonaute-1. Thus, automiG reports *in vivo* for both miRNA biogenesis and Ago-2 mediated silencing, providing a powerful biosensor to identify situations where miRNA or siRNA pathways are impaired. As a proof of concept, we used automiG as a biosensor to screen a chemical library and identified 29 molecules that strongly inhibit miRNA silencing, out of which 5 also inhibit RNAi triggered by long double-stranded RNA. Finally, the automiG sensor is also self-silenced by the anti-GFP miRNAs in HeLa cells and might be easily used to identify factors involved in miRNA biogenesis and silencing guided by perfect target complementarity in mammals.

## Introduction

Gene silencing by small interfering RNAs (siRNAs) and micro-RNAs (miRNAs) involves compartmentalized pathways in *Drosophila*. Perfectly base-paired 21 nt siRNA duplexes arise from the processing of long double-stranded (ds) RNAs by the Dicer-2 enzyme [1], [2] and are typically loaded into the Argonaute-2 protein [3], [4]. After unwinding, one strand of a siRNA duplex guides Ago-2 for the cleavage of RNA targets with extensive siRNA sequence complementarity, a process referred to as RNA interference (RNAi) [5]. siRNAs produced from viral RNA genomes have an essential role in antiviral defense, which is well illustrated by the dramatic sensibility of *Drosophila dcr2* or *ago2* mutants to viral infections [6]–[8]. In addition, siRNAs produced from dsRNA of endogenous origin (endo-siRNAs) play a role in transposon silencing [9]–[11] and heterochromatin formation [12].

MiRNAs derive from primary structured transcripts (pri-miRNAs) whose processing by the Drosha/Pasha microprocessor complex gives rise to precursor miRNAs (pre-miRNAs) with a typical ∼70 nt stem-loop structure. Cleavage of pre-miRNAs by the Dicer-1 enzyme removes the pre-miRNA loops and liberates ∼22 nt miRNA/miRNA* duplexes that, in contrast to siRNA duplexes, are mismatched at several positions [13]. Single-stranded mature miRNAs eventually guide Argonaute-1 for translational repression and/or destabilization of target mRNAs with partial complementarity in their 3′ UTR [5], [14]. miRNAs are important regulators of development and cell differentiation in metazoans [15], [16]. Consistently, there is a growing body of evidence that alterations in miRNA expression or activity are linked to cancers and genetic diseases [17]–[19].

Although the majority of *Drosophila* miRNAs are preferentially loaded into Ago1, a subset of miRNA preferentially associates with Ago2 [20], [21]. In addition miRNAs* strands, thus far considered as by-products of miRNA biogenesis, tend to accumulate in association with Ago2 [22]–[24]. Collectively, these results uncovered a new level of complexity in the miRNA-silencing pathway as well as partial overlap with the siRNA-silencing pathway.

We are interested in identifying *Drosophila* factors required for miRNA biogenesis or activity. Several systems to screen *in vivo* for genes involved in miRNA silencing in flies have been previously described. They relied on one vector expressing a miRNA plus one vector expressing a reporter gene engineered to carry the corresponding miRNA target in its 3′ UTR [25]–[27]. Within the context of high-throughput screens, such two-component systems may generate both false negative and false positive hits. For instance, down regulation of the miR expression vector may be associated to false positives whereas hits associated to low reporter signal may be discarded during signal background filtering. We reasoned that a single-component reporter system with a high dynamic range of response could circumvent these limitations. To this aim, we generated a single gene construct that simultaneously expresses the GFP as well as 2 artificial miRNAs perfectly matched to 2 distinct sites in the GFP coding sequence for maximizing GFP silencing. We showed that strong self-silencing of the resulting automiG gene involves the canonical miRNA biogenesis pathway as well as Ago2, thereby providing a highly dynamic biosensor of both miRNA biogenesis and Ago2-mediated silencing. To test its robustness and versatility, we used the automiG sensor in a chemical library screening and identified compounds that suppressed Ago2-mediated miRNA silencing. In addition, we showed that the automiG sensor might be easily used to identify factors involved in miRNA biogenesis or activity in human cells.

## Experimental Procedures

### Plasmid Constructs

**Figure 1:**
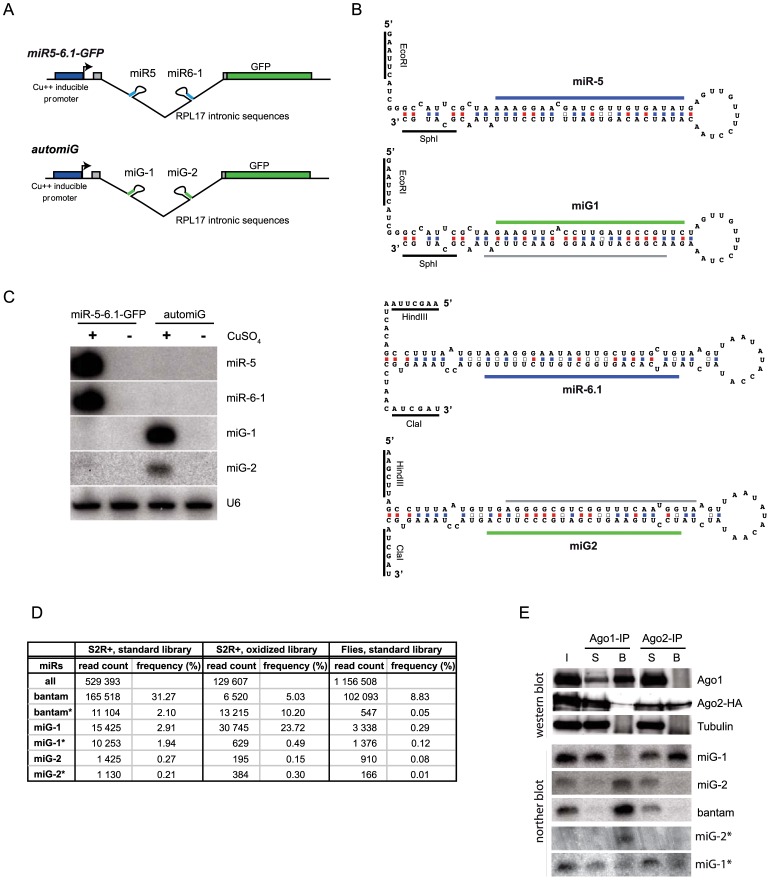
The automiG construct expresses miRNAs targeted to GFP sequences. (**A**) Schemes of the miR5-6.1-GFP and automiG constructs. (**B**) Folded sequences of miR-5, miR-6.1, miG-1 and miG-2 precursors in miR5-6.1-GFP or automiG constructs. Positions of mature miR-5 and miR-6.1 sequences are indicated with blue bars. Positions of mature miG-1 and miG-2 sequences are indicated with green bars. Positions of miG-1* and miG-2* sequences are indicated with grey bars. (**C**) S2R+ cell lines stably transfected with miR5-6.1-GFP or automiG were grown for 48 h in the presence or absence of CuSO_4_. Total RNA from these cells was analyzed in northern blot using the indicated radiolabeled probes. U6 RNA was used as a loading control. (**D**) Solexa sequencing of small RNA libraries prepared from S2R+ cells or transgenic flies stably transfected with the *ubi*-automiG construct. The oxidized library was generated from small RNA treated by NaIO_4_ before adapter ligation. For each library, the total number of reads of *Drosophila* miRNAs (all) is indicated, as well as the number of reads of bantam, miG-1, miG-2 and of the corresponding miR* species. Frequencies are expressed relatively to the total number of miRNA reads in the libraries. (**E**) S2 cells stably transfected with automiG and a construct expressing a tagged Ago2-HA protein were grown for 48 h in the presence of CuSO_4_. A total extract from these cells was split and immunoprecipitated using anti-Ago1 (Ago1-IP) or anti-HA (Ago-2-IP) antibodies. Protein content of input (I), supernatant (S) and bound fractions (B) was analyzed by western blot using the indicated antibodies. RNA was extracted from I, S and B fractions and analyzed by northern blot using miG-1, miG-2, miG-1*, miG-2* and bantam radiolabeled probes.

A Gateway pENTR-3C vector (Invitrogen) was engineered to give rise to pENTR-3C_miR5-miR6. This construct includes the exon2-intron2-exon3 region of the *RpL17* gene fused to the GFP coding sequences. We replaced a 262 bp region from the *RpL17* intron by a 262 bp genomic region containing mir-5 and mi-6-1 in which EcoRI, SphI, HindIII and ClaI sites were introduced to facilitate subsequent mir substitution. (plasmid map available upon request). A pENTR-3C_miG1_miG2 vector was then produced by replacing the EcoRI-mir-5-SphI and HindIII-mir-6-1-ClaI fragments in pENTR-3C_miR5-miR6 by EcoRI-miG1-SphI and HindIII-miG2-ClaI sequences, as depicted in Fig. 1A. Derivative constructs pENTR-3C_Δ1-miG2, pENTR-3C_Δ1–Δ2 and pENTR-3C_miG1-Δ2 were generated by restriction-mediated deletion of miG1, miG2 or both miG1 and miG2 segments. Finally, appropriate pENTR derivative vectors were recombined with the Gateway pDEST-48 destination vector for metallothionein promoter driven expression in *Drosophila* cells (Invitrogen) to give rise to the miR5-6.1-GFP, automiG, automiG-Δ1–2, automiG-1–Δ2 and automiG-Δ1–Δ2 constructs. The ubiquitin-automiG construct variant used for *Drosophila* transgenesis was generated by recombining the pENTR-3C_miG1–miG2 vector with the destination vector pUWG (a kind gift from Clara Moch and Jean-René Huynh) described at the Drosophila Genome Ressource Center (https://dgrc.cgb.indiana.edu/). pCI-neo mammalian expression vector (*Promega®*) was used as backbone vector to generate a Gateway compatible destination vector using the *Gateway® Vector Conversion System*. pCI-neo destination vector (a gift from Yves Jacob, Pasteur Institute) was then recombined with the previously described pENTR-3C_miG1_miG2 to give rise to the CMV-automiG vector driven automiG expression in mammalian cells. Sequences of all constructs were verified and are available upon request.

### Stable Cell Lines and Transient Transfections

Drosophila S2R+ (from DRSC, http://www.flyrnai.org/) and S2 cells (Invitrogen) were cultured in Schneider’s Drosophila medium (GIBCO-Invitrogen), supplemented with 10% heat-inactivated fetal calf serum, 100 U/ml penicillin and 100 µg/ml streptomycin. S2R+ stable lines transfected with miR5-6.1-GFP, automiG or automiG and Ago2-HA [9] were generated according to the Invitrogen *Drosophila* Expression System protocol using blasticidin as a selection marker (25 µg/ml, Invitrogen, Carlsbad, CA). Transfections of S2R+ and S2 cells were performed using Effecten transfection reagent (Qiagen) according to manufacturer’s instructions. The copper-inducible *metallothionein* promoter was induced by 600 µM CuSO_4_.

### Immunoprecipitations

The S2R+ cell line stably transfected with automiG and HA-Ago2 was harvested 20 h after induction by CuSO_4_, washed twice in PBS, and lysed on ice for 30 min in Lysis Buffer (50 mM Tris pH7.5, 150 mM NaCl, 2.5 mM MgCl2, 250 mM sucrose, 0.05% Nonidet P-40, 0.5% Triton X-100, 1 mM DTT, 1X protease inhibitor mixture and 40 U RNase OUT). Supernatant was incubated with anti-Ago1 (Abcam ab5070) or anti-HA (Roche) antibodies and equilibrated Gamma bind-Plus resin slurry (GE Healthcare) for 7 h at 4°C. Supernatant was kept for analysis and beads were washed 5 times in Lysis Buffer. 25% of the beads and 25% of the supernatant were used for western blot analysis. The remaining fractions of supernatant and beads were incubated 2 h with proteinase K, extracted with phenol-chloroform-isoamyl alcohol, precipitated with glycogen in isopropanol, washed in 80% ethanol, resuspended in water and analyzed by Northern blot.

### Immunoblot Analysis

For protein analysis, equal amounts of proteins from total extract, supernatant, or immunoprecipitate samples were boiled in Laemmli buffer and loaded on 12% SDS/PAGE. After transfer onto nitrocellulose membrane and Ponceau staining, membranes were blocked in 5% milk, 1X PBS, 0.1% Tween, and incubated overnight with anti-GFP (1∶2000; Roche), anti-Mbf1 (1∶10000; [28], anti-Ago1 (1∶1000; Abcam ab5070), anti-HA (1∶1000; Roche) or anti-γ-Tubulin (1∶2000; Sigma) antibodies. anti-Dcr-1, anti-Dcr-2 and anti-Ago2 were used at 1∶1000 (a kind gift of Mikiko C. Siomi). Appropriate secondary antibodies (1∶5000) coupled to HRP (GE Healthcare) or Alkaline phosphatase (Biorad) were added and incubated for 2 h at room temperature. Detection was performed using Super signal Chemiluminescent Substrate (Pierce) or the BCIP/NBT detection (Thermo Scientific).

### Northern Blot Analysis

For Northern blot analysis of miRNA, 10 µg of S2R+ total RNA extracted using Trizol Reagent (Invitrogen) and resuspended in water were resolved in 15% urea-polyacrylamide gel, transferred to Hybond-NX membrane (GE Healthcare) and UV-crosslinked. Oligonucleotides were 5′ ^32^P end-labeled using T4 polynucleotide kinase (Fermentas) and used as probes. Hybridization was performed over-night at 39°C (for miRNA) or 50°C (for U6) in PerfectHyb Plus (Sigma) hybridization buffer. Probes were AS-miR5 5′-CATATCACAACGATCGTTCCTTT-3′, AS-miR6 5′-AAAAAGAACAGCCACTGTGATA-3′, AS-miG1 5′-AGAACGGCATCAAGGTGAACTTC-3′, AS-miG2 5′-TGAAGGGCATCGACTTCAAGGA-3′, AS-miG1* 5′-ATGAAGTTCCCTTAATGCCGTT-3′, AS-miG2* 5′-TACCATTGAAACCGACGCCCCT-3′ AS-U6 5′-CGATTTTGCGTGTCATCCTTGC-3′. The bantam probe was previously described [29].

### Preparation of the Small RNA Libraries and High-throughput Sequencing

Total RNA was extracted using Trizol (Invitrogen) and submitted to Bioanalyser for quality assessment. Small RNAs from the S2R+ stable cell line or transgenic adult flies expressing the automiG construct were cloned using the DGE-Small RNA Sample Prep Kit and the Small RNA Sample Prep v1.5 Conversion Kit from Illumina, following the manufacturer’s instructions. The oxidized library was generated from small RNA treated by NaIO_4_ before adapter ligation as previously described [30]. Libraries were sequenced using the Illumina Genome Analyzer II. Sequence analysis and miRNA profiling were performed as previously described [31] using the *Drosophila melanogaster* miR database from miRBase (release r16) as a reference.

### RNA Interference

Double stranded RNAs were synthetized by *in vitro* transcription (Ambion MEGAscript® T7 Kit) of PCR products amplified from *w^1118^* genomic DNA primers flanked by T7 promoters. Sequences of amplicon templates for dsRNA production are available at the Drosophila RNAi Screening Center (http://www.flyrnai.org/cgi-bin/RNAi_gene_lookup_public.pl) and identified as follows: Drosha DRSC07607, Dcr-1 DRSC27421, Dcr-2 DRSC29436, Ago1 DRSC05912, Ago2 DRSC10847 and DRSC31768. PCR products for T7 transcription of Firefly Luciferase or GFP dsRNAs were amplified using the following primers: 5′-TAATACGACTCACTATAGGGATGCACATATCGAGGTGGAC-3′ and 5′-TAATACGACTCACTATAGGGAGAATCTCACGCAGGCAGTTC-3′ (Luciferase), 5′-GAATTGTAATACGACTCACTATAGGGCTTACTTGTACAGCTCGTC-3′ and 5′-GAATTGTAATACGACTCACTATAGGGCATGGTGAGCAAGGGCGAG-3′ (GFP). S2R+ cells were cultured at a concentration of 10^6^ cells/ml and incubated with 6 µg/ml of dsRNA. S2 cells were cultured in 24-well plate and transfected with 6 ng of dsRNA using Effecten transfection reagent (Qiagen, Hilden) according to manufacturer’s instructions.

HeLa cells were co-transfected with the indicated siRNA and the CMV-automiG vector using Thermo Scientific*® DharmaFECT Duo Transfection Reagent* following the manufacturer protocol. Sequences of siRNAs were previously described [32]. After 72 hours, culture medium was carefully removed and 100 µl of cracking buffer (125 mM Tris pH6,8, 5% ß-mercapto-ethanol, 2% SDS, 4 M Urea) was added in each well. 20 µl of each protein extracts were analyzed by western-blot using anti-GFP and anti-γ-Tubulin (loading control) antibodies.

### Chemical Library Screening

We screened 1120 compounds purchased from the Prestwick chemical library (http://www.prestwickchemical.com/) and 9360 compounds (ATP-mimic library) and 4624 compounds (nucleoside-like library) from ChemDiv Inc (http://eu.chemdiv.com/). Most of those molecules can also be purchased individually by browsing commercial online databases using the indicated reference numbers or structures. Further ordering information is available upon request. All molecules were dissolved in dimethylsulfoxide (DMSO) and used at a concentration of 5 to 10 mM. Thanks to a pipetting head (TECAN), 100 µl of automiG S2R+ cells at a concentration of 10^6^ cells/ml in culture medium containing 600 µM CuSO_4_ were distributed in 96-well plates containing in each well 1 µl of compound in DMSO. Columns 1 and 12 were dedicated to negative controls (DMSO alone). After incubation for 2 days at 25°C, GFP fluorescence was measured using a Safire^2^™ (TECAN) microplate reader. Fold-changes in GFP expression were calculated from the ratio of fluorescence measured in individual wells to the mean of fluorescence measured for negative controls. We also used an automated Nikon TE 2000 inverted microscope connected to a Nikon camera with QUIA and Lucia software to capture and store images from plate wells at 40×magnification. For each well, informatic image analysis was performed to determine the number of fluorescent spots in one microscope field. A validation screen was performed in triplicate using the same conditions as those used in the primary screen. After the 48 h incubation period, plates were centrifuged 1 min at 800 g and the culture medium was carefully removed. 25 µl of cracking buffer (125 mM Tris pH6,8, 5% ß-mercapto-ethanol, 2% SDS, 4 M urea) was added in each well and 8 µl of protein extract were analyzed by western-blot using anti-GFP antibody.

To test the effect of the validated compounds on RNAi triggered by long dsRNA, 100 µl of automiG-Δ1–Δ2 S2R+ cells (10^6^ cells/ml) were dispensed in wells of a 96-well plate supplemented with 6 µg/ml of GFP dsRNA and 1 µl of chemical in DMSO (or 1 µl of DMSO for negative controls) and incubated for 24 h at 25°C. Six µl of a 10 mM CuSO_4_ solution were then added in each well and cells were incubated for additional 24 h. GFP fluorescence was measured using the plate-reader just before and 24 h after copper induction. Proteins were extracted and analyzed by western-blot for GFP expression 24 h after copper induction.

To test the effect of compounds on human miRNA pathway, HeLa cells were transfected in 24-well plates with the CMV-automiG using Thermo Scientific*® DharmaFECT Duo Transfection Reagent* following the provider protocol. Twenty-four hours after, chemicals used at a final concentration of 30 to 50 µM or DMSO alone (control) were added to the medium. After an additional 48 h period, protein extraction was performed. Culture medium was carefully removed and 100 µl of cracking buffer (125 mM Tris pH 6.8, 5% ß-mercapto-ethanol, 2% SDS, 4 M urea) was added in each well. 20 µl of protein extract were analyzed by western-blot using anti-GFP and anti- γ -Tubulin (loading control) antibodies.

### Immunofluorescence of Imaginal Discs

Wing discs from third-instar larvae were dissected in 1X cold PBS and fixed in PBS with 4% paraformaldehyde for 20 min at 4°C. The disks were washed with PBS 3 times 10 min, blocked in 1X PBS, 0.05% Tween 20, 0.2% bovine serum albumin for 30 min, and stained with anti-GFP antibody (Roche) overnight. After 3 washes of 10 min in PBST 0.05% discs were incubated with the secondary antibody (anti-mouse coupled to FITC (Jackson laboratory)) 2 hours at room temperature. After 3 washes of 10 min in PBST, discs were transferred to PBS 1X and mounted in Progold-DAPI mounting medium.

### Fly Stocks

Flies transgenic for the ubiquitin-automiG and UASp-automiG constructs were established using P-element mediated transformation (Bestgene Inc). Dcr-2^R416X^ [1] and AGO2^414^ [4] mutant stocks were previously described. UAS-IR[Ago1]; y1 v1; P{TRiP.HM04006}attP2 (#31700) and UAS-IR[Ago2] (#49473) were ordered at the Bloomington and VDRC stock centers respectively.

## Results and Discussion

### Design of the AutomiG Sensor

A region encompassing the first coding nucleotides of exon-2, the second intron, and the first nucleotide of exon-3 from the Drosophila *RpL17* gene (CG3203) was fused to the coding sequence of the GFP and put under the control of the copper-inducible metallothionein promoter (Fig. 1A). Within the *RpL17* intron, we inserted a fragment of a miRNA cluster encompassing the pre-miR-5 and pre-miR-6-1 sequences, each flanked by cloning sites to facilitate further mutagenesis (Fig. 1B). *Drosophila* miR-5 and miR6.1 are not expressed in S2R+ cultured cells. In contrast, S2R+ cells stably transfected with the miR5-6.1-GFP construct express mature miR-5 and miR-6.1 miRNAs upon copper induction (Fig. 1C).

We next replaced the miR-5 and miR-6-1 pre-miRNA sequences by synthetic miG-1 and miG-2 pre-miRNA sequences, respectively, so that expected mature miG-1 and miG-2 miRNAs match two distinct targets in the GFP coding sequence with perfect complementarity (Fig. 1B). Bulges and mismatches were introduced in the stems of pre-miG-1 and pre-miG-2 to keep as much as possible the pre-miR-5 and pre-miR-6.1 backbone structures. Upon copper induction, S2R+ cells stably transfected with the resulting automiG construct expressed miG-1 and, to a lesser extent, miG-2 (Fig. 1C). The change in the relative abundance of miG-1 and miG-2, as compared to the relative abundance of miR-5 and miR-6 (Fig. 1C) prompted us to analyze in further detail whether reprograming of the automiG construct affected the biogenesis of the artificial miRNAs. Solexa sequencing of a small RNA library prepared from the automiG S2R+ cells indicated that miG-1 and miG-2 rank among the more expressed miRNAs and revealed the presence of miG-1* and miG-2* species (Fig. 1D and Table S1). Predominant forms of mature miG-1, miG-2, miG-1* and miG-2* were 22 nt long and mapped at their expected positions in the pre-miG-1 and pre-miG-2 backbones (Fig. 1B and data not shown), indicating that reprogramming miR-5 and miR-6-1 into miG-1 and miG-2, respectively, affected the relative steady state levels of the artificial miRNAs, but did not affect the precision of their processing by Drosha and Dicer-1.

To determine how miG-1 and miG-2 partition between Ago1 and Ago2 silencing complexes, we generated a S2R+ cell line stably transfected with both the automiG construct and a HA-tagged Ago2 construct under the control of its endogenous promoter [9]. Small RNAs from this cell line were co-immunoprecipitated either with Ago1 or with Ago2-HA and analyzed by northern blotting using miG-1, miG-2, miG-1*, miG2* and bantam probes (Fig. 1E). The bantam miRNA predominantly associates with Ago1 [4], [9], [20]. In agreement, we found bantam almost exclusively associated to Ago1. Similarly, miG-2 was mainly recovered in Ago1 eluate (Fig. 1E), although a small fraction of miG2 bound to Ago2 could be revealed in independent experiments over longer exposure (data not shown). In striking contrast, miG-1 was almost exclusively recovered in Ago2 IP product. Because miRNA star species have been shown also associated to Ago1 or Ago2 [22]–[24], we probed for the association of the miG* species to these argonautes proteins. miG-2* appeared mostly associated to Ago1, whereas miG-1* distributed between Ago1 and Ago2 (Fig. 1E).

Small RNAs loaded in Ago2 are 2′-O-methylated at their 3′ ends [33]–[35], a modification that protects them from oxidation by NaIO_4_ treatment. In contrast, miRNAs loaded in Ago1 are unmethylated and accessible to oxidation by NaIO_4_. We prepared another small RNA library from copper-induced automiG cells using oxidation by NaIO4 prior to the adapter ligation [30]. Solexa sequencing revealed that miG-1 is dramatically enriched in this library as compared to the non-oxidized library (23.72% vs 2.91%), agreeing with its loading in Ago2 (Fig. 1D, Table S1). Consistently with the partial loading of miG-1* in Ago1 (Fig. 1E), miG-1* was less abundant in the oxidized library (0.49% vs 1.94%). As expected from their preferential loading in Ago1, bantam (5.03% vs 31.27%) and miG-2 (0.15% vs 0.27%) relative abundances were reduced in the oxidized library. miG-2* was not significantly enriched in the oxidized library (0.30% vs 0.21%), agreeing with its preferential loading in Ago1.

Altogether, these experiments pointed to a differential partitioning of miG-1 and miG-2 in Argonaute silencing complexes that is indeed in agreement with sorting rules deduced from previous studies [22]–[24]. On the one hand, nucleotides at position 9 and 10 relative to the 5′ end of miG-2 show wobble pairing in the miG-2/miG-2* duplex and miG-2 starts with a 5′ U (Fig. 1B). These features, which are observed for a majority of miRNAs, make it more suitable for Ago1 loading. On the other hand, nucleotides 1, 2 and 9 to 11 relative to the 5′ end of miG-1 show a perfect pairing in the miG-1/miG-1* duplex and miG-1 does not start with a uridine. These features, which are typical of siRNAs, make it more suitable for Ago2 loading. Interestingly, miR-5 starts with a uridine and has a wobble pairing in position 9 which is perfectly paired at the corresponding position in the miG-1/miG-1* duplex. Moreover, our analysis of available small RNA sequence datasets [23] suggests a preferential miR-5 loading in Ago1 in drosophila heads (not shown). Thus, changing the first nucleotide identity and the pairing at position 9 in the miR duplex for reprogramming miR-5 into miG-1 may have been sufficient to redirect miG-1 to Ago2. It is also interesting to note that the partitioning of the miG* species is not reciprocal to the partitioning of their miG counterparts, as miG-2* and, to a lesser extent, miG-1* are both loaded in Ago1. This observation supports the notion of independent sorting of *Drosophila* miRNA duplex strands in Ago proteins [24].

### Characterization of the AutomiG Silencing

**Figure 2:**
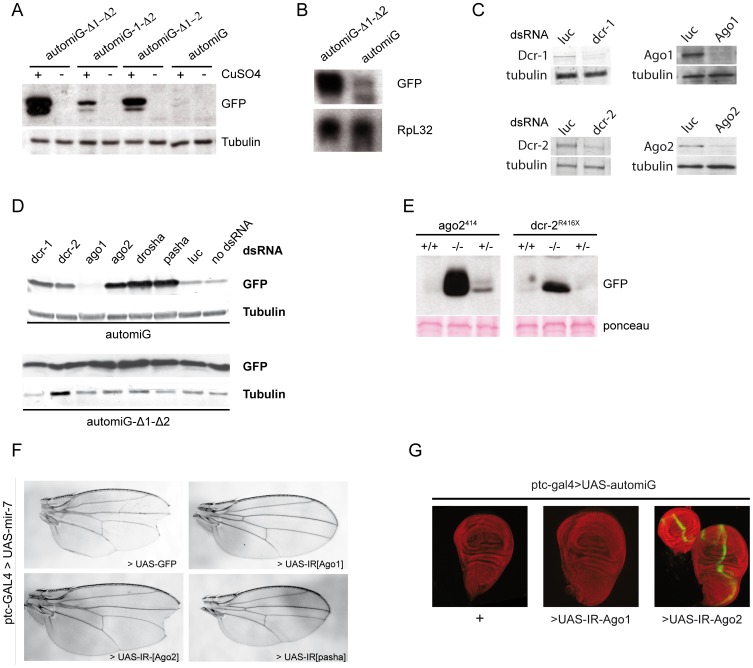
The automiG construct is a sensor of Ago2-mediated miRNA silencing. (**A**) S2R+ cell lines stably transfected with automiG, automiG-Δ1–2, automiG-1–Δ2 and automiG-Δ1–Δ2 constructs were grown for 48 h in the presence or absence of CuSO_4_, as indicated and GFP expression was analyzed by western blotting. (**B**) GFP mRNAs are degraded in automiG expressing cells. S2 cells transformed with automiG or automiG-Δ1–Δ2 constructs were grown for 24 h in the presence of CuSO4. Total RNA was extracted and analyzed by northern blot using GFP and RpL32 radiolabeled probes as indicated. (**C**) Control of dsRNA efficiency. The automiG S2R+ stable cell line was soaked for 48 h with the indicated dsRNA, induced by CuSO_4_ for 6 additional hours and expression of Dcr-1, Dcr-2, Ago1 and Ago2 was analyzed by western blot. The γ-Tubulin protein was used as a loading control. (**D**) automiG S2R+ and automiG-Δ1–Δ2 stable cell lines were soaked for 48 h with the indicated dsRNA, induced by CuSO_4_ for 6 additional hours and GFP expression was analyzed by western blot. The γ-Tubulin protein was used as a loading control. (**E**) GFP expression from the *ubi*-automiG transgene in wild type, heterozygous or homozygous *ago2^414^* or *dcr2^R416X^* adult flies was analyzed by western blotting. Ponceau staining was used to control equal loading of the lanes. (**F**) Ago1 RNAi in the patch territory suppressed the Ago1-dependent miR-7 effect in the wings. Wings from ptc-GAL4>UAS-mir-7 adult flies in the presence of the UAS-GFP transgene as a control or of the hairpin transgenes UAS-IR[Ago1], UAS-IR[Ago2] or UAS-IR[pasha] are shown. Overexpression of mir-7 in the patch territory induces a distal wing notch in control flies, that is suppressed by Ago1 and pasha RNAi knockdowns but not by Ago2 RNAi knockdown. (**G**) GFP (green) expression in wing discs from ptc-GAL4, UASp-automiG/UAS-IR[Ago1] or ptc-GAL4, UASp-automiG/UAS-IR[Ago2] third-instar larvae is shown. Control discs ptc-GAL4, UASp-automiG/+ did not induce a significant GFP signal in control experiments. DAPI staining is shown in red.

The GFP protein (Fig. 2A) as well as the GFP mRNA (Fig. 2B) were dramatically down-regulated in copper-induced automiG cells as compared to cells transfected with an automiG-Δ1–Δ2 control construct in which both pre-miG-1 and pre-miG-2 sequences are deleted, demonstrating a strong self-silencing of the automiG construct by the reprogrammed miG-1 and miG-2 miRNAs. GFP expression in a cell line transfected with the automiG-1–Δ2 construct in which only the pre-miG2 sequence is deleted was very modest, whereas it was intermediate in a cell line transfected with an automiG-Δ1–2 cell line in which only the pre-miG-1 sequence is deleted (Fig. 2A). Copy numbers of stably transfected constructs may vary between cell lines. Hence, it is possible that GFP expression levels do not accurately reflect the relative strengths of self-silencing of the automiG variants. Nevertheless, the data suggest that miG-1 predominantly associated to Ago2 contributes more to the net silencing of the automiG construct than miG-2 predominantly associated to Ago1. This conclusion is fully supported by the previous finding that the efficiency of the high turn-over Ago2 enzyme exceeds the one of Ago1 by one order of magnitude for silencing targets with perfect base complementarity [21].

To further characterize the automiG self-repression, S2R+ cell lines transfected with the automiG construct or with the control automiG-Δ1–Δ2 construct were incubated for 2 days with dsRNA targeting key components of small RNA silencing pathways. When available, RNAi knockdown efficiencies were assayed at the protein level using appropriate antibodies (Fig. 2C). The automiG and automiG-Δ1–Δ2 constructs were then induced with copper and GFP expression was monitored 6 h later by western blot assay.

The automiG-Δ1–Δ2 control construct expressed high levels of GFP under copper induction and none of the tested dsRNAs had a significant effect on GFP expression (Fig. 2D). As expected, the automiG construct expressed only very low level of GFP in the absence of dsRNA (no dsRNA) or in the presence of control luciferase dsRNA (luc). In striking contrast, RNAi depletion of Drosha, of its cofactor Pasha or Dicer1 significantly induced GFP expression by automiG as compared to the controls, indicating that miG-1 and miG-2 biogenesis involves the canonical components required for primary and pre-miRNA processing. Dicer2 and Ago2 depletion induced a dramatic increase in GFP expression. Furthermore, GFP expression was barely detectable in flies transgenic for a *ubi*-automiG variant under the control of the ubiquitin-63E promoter (Fig. 2E), whereas it was strongly induced in *ubi*-automiG transgenic flies homozygous for the *ago2^414^* and *dcr2^R416X^* mutations (Fig. 2E). Together, the data indicate that miG-1 and miG-2, as shown for a subset of miRNAs, exerts their silencing activity through the RNAi enzyme Ago2. It is noteworthy that, in the Ago2-dependent small RNA silencing pathway, Dcr2 in association with R2D2 plays a critical role in the loading of small RNA duplexes independently of it dsRNA dicing activity [20], [36].

In contrast to Ago2, Ago1 dsRNA treatment did not relieve and even weakly enhanced the automiG silencing in S2R+ cells (Fig. 2D, compare with control dsRNA). This result suggests that Ago1 could sequester miG2 or other factors involved in Ago2-mediated silencing and indicates that both miG-1 and miG-2 function exclusively through Ago2.

To further support the exclusive contribution of Ago2 to the automiG silencing, we turned toward *in vivo* RNAi. Thus, we triggered Ago1 RNAi in wing imaginal discs using a UAS-Ago1 hairpin transgene under the control of a ptc>GAL4 driver transgene. The silencing efficiency of Ago1 RNAi in the *patch* territory was demonstrated by the suppression of the Ago1-dependent miR-7 effect in the wing (Fig. 2F and [37]. Nonetheless, under the same Ago1 RNAi condition, we could not detect GFP expression from a *UAS*-automiG variant transgene in the *patch* territory (Fig. 2G). In striking contrast, UAS-automiG was desilenced under Ago2 RNAi.

In summary, the automiG construct expresses two miRNAs miG-1 and miG-2 whose biogenesis involves Drosha, Pasha, Dicer-1 and Dicer-2. Silencing of automiG appears to exclusively involve Ago2 loaded with miG-1 and, to a lower extent, with miG-2. The enhanced silencing of AutomiG observed upon Ago1 depletion in S2R+ cells may be due to the release of miG2 or other Ago1 cofactors also involved in Ago2-mediated silencing.

### AutomiG is Suitable for High-throughput Screening Approaches

MicroRNAs have been linked to a variety of diseases including cancers [38]. Thus, identification of small molecules able to activate tumor-suppressive miRNAs or inhibit oncogenic miRNAs provided new approach for the development of cancer therapeutics [39]–[44]. This context prompted us to test the robustness and the sensibility of the automiG sensor in a high throughput screening for molecules that suppress miRNA biogenesis or Ago2-mediated miRNA silencing.

**Figure 3:**
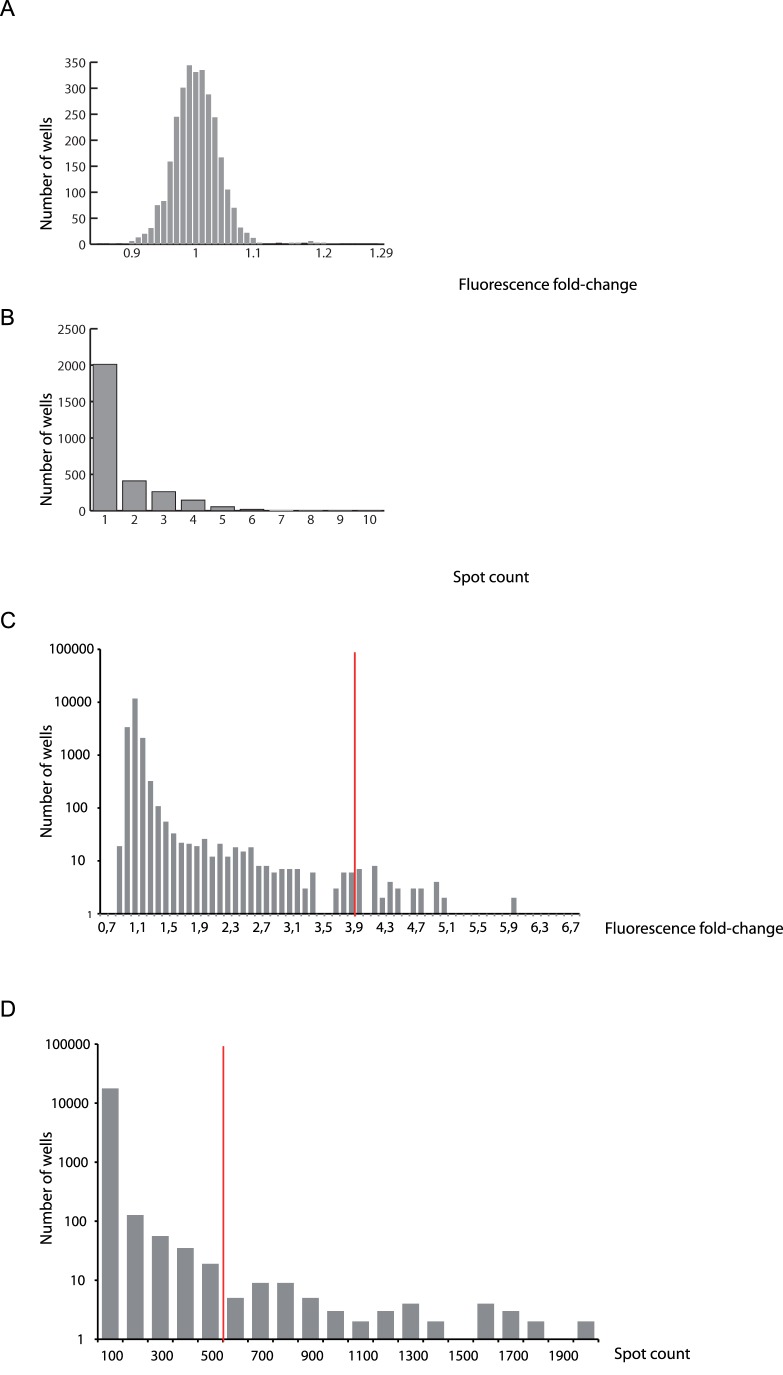
Screening of chemical compounds using the automiG construct. Fluorescence fold-change (**A**) and fluorescent spot count (**B**) distributions in negative controls with DMSO only. Fluorescence fold-change (**C**) and fluorescent spot count (**D**) distributions in wells with compounds from the chemical libraries. Note the logarithmic scales for these distributions. Red lines correspond to the cut-offs used for selection of the compounds.

**Figure 4:**
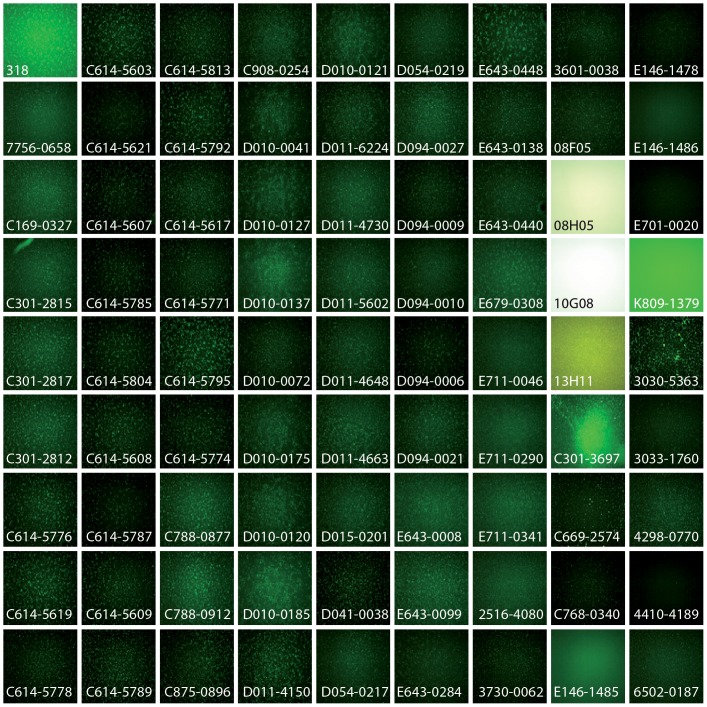
81 compounds induce strong fluorescence of automiG cells. The primary screen identified 46 compounds that induced a fluorescence fold-change >3.9 and 57 compound for which more that 500 fluorescent spots were counted from well imaging (see materials and methods). The merging of these two sets yield 81 compounds whose effects on automiG cells are presented. Each panel correspond to one image taken by the automated Nikon TE2000 inverted microscope. Note that out of the 81 compounds, 17 were discarded as false positives because they induced by themselves fluorescence (see Supporting Information Table S2, shaded identifiers).

S2R+ cells stably transfected with the automiG sensor were bathed in 96-well plate containing copper sulfate and 15,104 commercially available compounds. Two days later, induction of automiG expression was calculated as the ratio of fluorescence measured in individual wells to the mean fluorescence of negative controls included in each plate. Using an automated microscope we also acquired images of the wells. AutomiG induction level was calculated from these images using an in-house software that determines the number of fluorescent spots in one microscope field at 40×magnification (spot count). Fluorescence fold changes and spot counts were combined to identify compounds that strongly suppress automiG silencing. It is noteworthy that the positive GFP readout provided by the automiG sensor eliminated compounds that impair cell viability. We selected a set of 46 compounds with a fluorescence fold-change above 3.9 and a set of 57 compounds for which more than 500 fluorescent spots were counted from well imaging (Fig. 3). Both sets overlapped and merging them yielded a list of 81 hit compounds. Microscope images corresponding to these compounds were then examined one by one (Fig. 4). Out of 81, 17 compounds were discarded as false positives because they induced by themselves diffuse fluorescence of the culture medium and/or the formation of large fluorescent crystals (Table S2, shaded identifiers).

**Figure 5:**
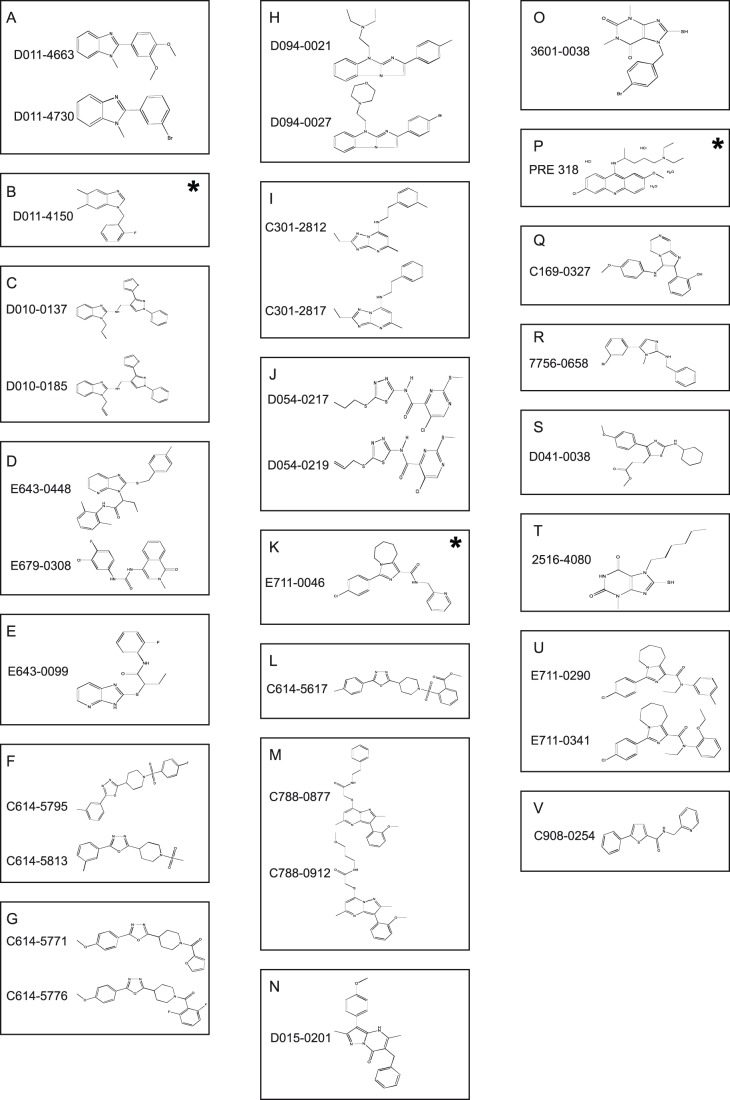
Structure of the molecules identified as inhibitors of automiG silencing in the primary chemical screening. Molecules are clustered in families (capitals A to V) using the Tanimoto coefficient and the K-modes procedure [46]–[48]. Molecules that were not validated in the secondary screen are indicated with an asterisk.

**Figure 6:**
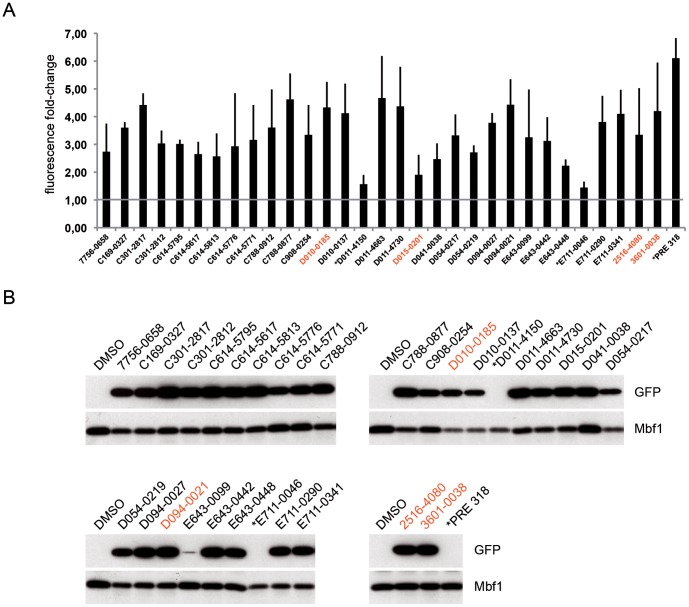
Suppression of the automiG silencing by hit compounds. Stably transfected automiG cells were grown for 24 h in the presence of CuSO_4_ and the indicated compounds. Fluorescence fold changes (**A**) were measured in triplicate experiments and GFP expression (**B**) was analyzed by western blot. Thin bars indicate the standard deviation. Red compound identifiers refer to compounds tested in HeLa cells (Fig. 8).

The 64 remaining molecules were clustered according to their structure in 22 chemical families (Table S2 and Fig. 5). One or 2 compounds of each family were re-tested in 96-well plates using a fluorescence plate reader in three independent trials (Fig. 6A). In addition, the effect of these molecules on GFP expression in automiG cells was directly assessed by western-blot (Fig. 6B). In both these assays, the effect of 2 compounds (D011-4150 and E711-0046) could not be reproduced. A third compound (PRE318) was associated to a significant fluorescence fold-change of automiG cells, but did not induce GFP expression in the western blot assay. Indeed, this compound becomes fluorescent when it was taken up by the cells (data not shown). At final, our pilot screen validated 29 molecules that strongly suppress the automiG silencing.

### A subset of Identified Compounds also Blocks RNAi Triggered by Long dsRNA

**Figure 7:**
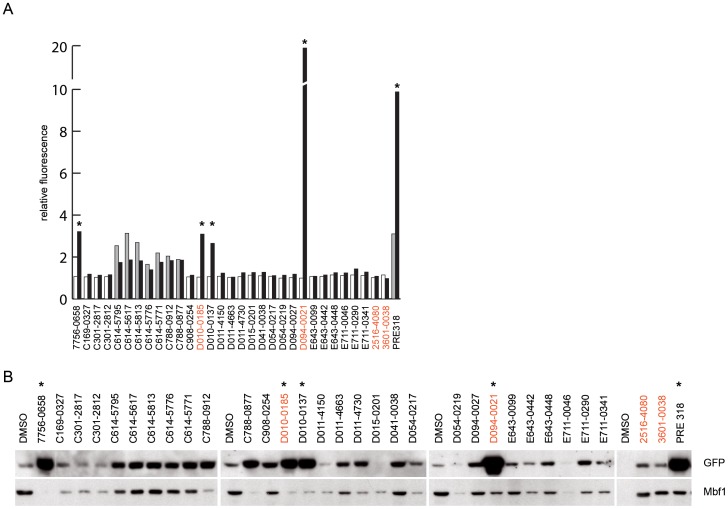
Suppression of RNA interference by hit compounds. S2R+ stably transfected with the automiG-Δ1–Δ2 construct were incubated for 24 h with the indicated compounds and GFP dsRNA. GFP expression was then induced by CuSO_4_ and cells were grown for further 24 h. Fluorescence relative to DMSO controls (**A**) was measured just before (white and dark grey bars) and 24 h after copper induction (black bars). GFP expression levels (**B**) were analyzed by western blot 24 h after copper induction. Asterisks point to compounds that inhibit RNAi. Note that compounds C614–5795 to C788–0877 triggered GFP expression before copper induction; although they were accordingly associated to high level of GFP, they were not marked as RNAi inhibitors. Red compound identifiers refer to compounds tested in HeLa cells (Fig. 8).

As Ago2 is the effector of the RNAi pathway, we also tested whether the compounds identified using the automiG sensor inhibit RNA interference triggered by long dsRNAs. To this aim, automiG-Δ1–Δ2 S2R+ cells were bathed for 24 h with both chemicals and dsRNA targeting the GFP coding sequence. Although the cells were grown in the absence of CuSO_4_ during this period of time, 8 of 32 compounds caused a significant increase of fluorescence relative to the DMSO controls (Fig. 7A, grey bars). This was expected from compound PRE318 that emits fluorescence when it is taken up into S2R+ cells (see above). The 7 other compounds may be contaminated with heavy divalent cations that would induce the expression of the GFP protein before copper induction. After additional 24 h in the presence of CuSO_4_, fluorescence relative to DMSO controls was stable or even decreased in cells treated by a majority of the compounds (Fig. 7A, black bars). In contrast, strong increase in fluorescence as well as a high level of GFP was detected for 5 compounds (Fig. 7, stars), indicating that they interfered with GFP RNAi.

### AutomiG Reports for miRNA Biogenesis in Mammalian Cells

**Figure 8:**
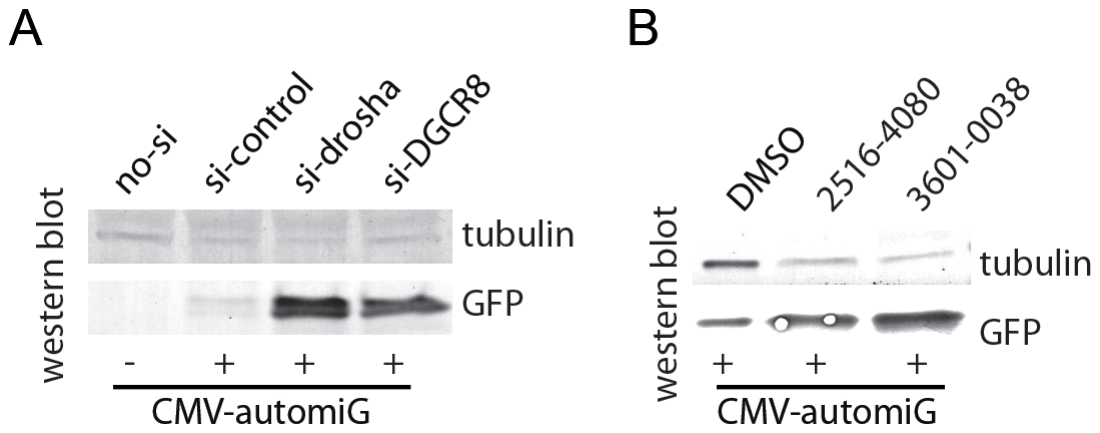
The CMV-automiG construct is a sensor of the human miRNA pathway. (**A**) HeLa cells transiently co-transfected with CMV-automiG and the indicated siRNAs were grown for 72 h and GFP expression was analyzed by western blot. The γ-Tubulin protein was used as a loading control. (**B**) Suppression of the CMV-automiG silencing by the #2516-4080 and #3601-0038 compounds. HeLa cells transiently transfected with CMV-automiG for 24 h were soaked for 48 additional hours with the indicated compounds or DMSO alone as a control, and GFP expression was analyzed by western blot. The γ-Tubulin protein was used as a loading control. The effects of the compounds #D010-0185 and #D094-0021 on GFP expression could not be assayed due to their toxicity.

As the mechanisms of both miRNA biogenesis and intron splicing appear to be conserved throughout evolution, the automiG construct could also provide a sensor system for miRNA biogenesis and activity in mammals. To test this possibility, we generated a CMV-automiG variant construct under the control of the CMV promoter which drives transcription in mammalian cell. When transfected alone or cotransfected with a scramble siRNA control in HeLa cells, the CMV-automiG construct expressed barely detectable levels of GFP protein (Fig. 8A). In striking contrast, cotransfections with siRNAs targeting the human key components of miRNA biogenesis, Drosha and its cofactor DGCR8, were associated with significant GFP induction (Fig. 8A), strongly suggesting that the CMV-automiG construct is indeed a biosensor for miRNA biogenesis in mammals. Finally, we selected the compounds #2516-4080 and #3601-0038, which inhibit miRNA silencing but not RNAi in S2R+ *Drosophila* cells, and the compounds #D010-0185 and #D094-0021, which inhibit both silencing pathways (Fig. 6 and 7), and we tested their effects on the CMV-automiG in HeLa cells. Twenty-four hours after transfection of HeLa cells with the CMV-automiG construct, the compounds were added to the medium for 48 h. The effect of the compounds #D010-0185 and #D094-0021 on GFP expression could not be assayed due to their toxicity (data not shown). In contrast, the compounds #2516-4080 and #3601-0038 were associated with significant GFP induction as compared to the DMSO control (Fig. 8B). The finding that compounds desilencing the automiG construct in *Drosophila* cells also desilence the CMV-automiG variant in HeLa cells further validates automiG as a versatile biosensor for miRNA biogenesis and activity.

### Conclusions

We engineered a robust and sensitive reporter system to study miRNA biogenesis and activity and we provided a proof of principle of its use in high throughput screening approaches. The system relies on an inducible gene construct expressing the GFP fluorescent protein together with miG-1 and miG-2, two intronic artificial miRNAs targeting the GFP mRNA. As intron splicing and Drosha cropping of intronic miRNAs do not interfere with each other [45], the ratio between miG1–miG2 miRNAs and the spliced GFP target RNAs, all produced from the same primary transcript by the automiG system, is most likely constant under normal conditions. Although we did not test in detail this assumption, we observed that the complete self silencing of the automiG reporter system is indeed insensitive to copy number variations upon transient cell transfections or in stably transfected cell lines. In addition, failure in intron splicing of the automiG transcript disrupts the open reading frame for the GFP reporter protein; should this event happen in screening approaches, it would not increase GFP expression and consequent false positive scoring. Finally, the presence of pools of miG-1 and miG-2 miRNAs already produced and engaged in the silencing machinery prior genetic or chemical disruption of miRNA/Ago2 silencing pathway could minimize the GFP desilencing in some instances. To circumvent this potential loss in sensitivity, miG-1, miG-2 as well as the GFP mRNA target have been put under the control of an inducible promoter. Thus, the automiG reporter system can be interrogated after genetic or chemical inactivation trials, which make it more sensitive to detect failures in miRNA/Ago2 silencing pathway. The dark side of this medal is that strong self-silencing of automiG in normal condition makes it less suitable for detecting enhancement of miRNA silencing. Nevertheless, AutomiG-Δ1–2 that is less repressed under normal condition (Fig. 2A) should be adapted to the detection of this effect.

The deep characterization of the automiG silencing revealed features fully consistent with the established connections between siRNA and miRNA pathways in *Drosophila*. Testing the automiG sensor in high-throughput conditions allowed us to identify 29 compounds that strongly inhibit Ago-2 mediated miRNA silencing. Five out of these compounds also inhibit RNAi triggered by long dsRNA, suggesting that they target steps common to both silencing mechanisms, such as RISC-Ago2 loading (RLC), unwinding of the small RNAs duplexes or target cleavage by RISC-Ago2. The other 25 compounds appear to specifically inhibit miRNA silencing. Further analyses are required to determine whether these compounds interfere with miRNA biogenesis, or specifically inhibit a miRNA loaded Ago2-RISC complex.

AutomiG provides a highly sensitive and accurate sensor system well adapted to high throughput screening approaches. Thus, in a genome-wide RNAi screen using an automiG cell line (manuscript in preparation), we recovered expected key genes of RNA silencing, including Ago2, Pasha and Drosha. In addition, we demonstrate here that the automiG system is versatile as it can be adapted to *Drosophila* cell lines or *Drosophila* transgenics as well as to human cells. Thus, our validation of the effect of two chemical compounds in CMV-automiG HeLa cells attests that the system has the potential to identify therapeutic drugs in miRNA-linked diseases.

## Supporting Information

Table S1 Abundances of miRNA sequencing reads in small RNA libraries from automiG S2R+ cells and from *ubi*-automiG transgenic adult flies. miG-1 and miG-2 are orange shaded. mir-5 and mir-6.1 are light-blue shaded. Note that the mir-5/mir-6 cluster is mostly expressed during embryogenesis and that only very few reads matching these miRNAs are recovered from the S2R+ and adult flies libraries.
(XLS)

Table S2 List of selected compounds. The compounds were clustered in 22 structural families (A to V) using the Tanimoto coefficient and the K-modes procedure. Compounds selected for further validation are enlightened in orange. Shaded compounds were discarded as false positives after microscopy analysis because they emit diffuse fluorescence in the culture medium and/or form large fluorescent crystals.
(XLS)
